# Exploring Team Passing Networks and Player Movement Dynamics in Youth Association Football

**DOI:** 10.1371/journal.pone.0171156

**Published:** 2017-01-31

**Authors:** Bruno Gonçalves, Diogo Coutinho, Sara Santos, Carlos Lago-Penas, Sergio Jiménez, Jaime Sampaio

**Affiliations:** 1 Research Center in Sports Sciences, Health Sciences and Human Development (CIDESD), CreativeLab Research Community, Universidade de Trás-os-Montes e Alto Douro, Vila Real, Portugal; 2 Faculty of Education and Sport, University of Vigo, Pontevedra, Spain; 3 Facultad de Ciencias de la Actividad Física y el Deporte, Universidad Europea de Madrid, Madrid, Spain; University of Texas at Austin, UNITED STATES

## Abstract

Understanding how youth football players base their game interactions may constitute a solid criterion for fine-tuning the training process and, ultimately, to achieve better individual and team performances during competition. The present study aims to explore how passing networks and positioning variables can be linked to the match outcome in youth elite association football. The participants included 44 male elite players from under-15 and under-17 age groups. A passing network approach within positioning-derived variables was computed to identify the contributions of individual players for the overall team behaviour outcome during a simulated match. Results suggested that lower team passing dependency for a given player (expressed by lower betweenness network centrality scores) and high intra-team well-connected passing relations (expressed by higher closeness network centrality scores) were related to better outcomes. The correlation between the dyads’ positioning regularity and the passing density showed a most likely higher correlation in under-15 (moderate effect), indicating a possible more dependence of the ball position rather than in the under-17 teams (small/unclear effects). Overall, this study emphasizes the potential of coupling notational analyses with spatial-temporal relations to produce a more functional and holistic understanding of teams’ sports performance. Also, the social network analysis allowed to reveal novel key determinants of collective performance.

## Introduction

The assessment of performance determinants plays an important role in sports sciences, since the derived information will contribute to improve and expand the coaching process. Notational analysis has been presented as powerful framework to produce valid and reliable description of teams’ performance [1–3]. For example, variables such as the number of shots and successful passes have been used to describe why some teams are more successful than others in association football [4–7]. These previous studies reported that teams who present better values for the considered performance indicators may increase the opportunity to score and, ultimately, to win the matches.

Recently, the passing variables have been used to create social networks that represent relationships established by the teammates, with the aim to understand how the collective performance may be optimized [8–11]. Yamamoto and Yokoyama [12] showed that stochastically switched dynamics of the players throughout the game is mainly based on the team’ ability to self-organize according to the teammates’ behaviours. Cintia and colleagues [13] showed how data-driven approach may present a big potential to accurately predict the team success. Indeed, the authors demonstrated that a complex systems’ view on football passing data has the potential of revealing hidden behaviours and patterns. Within this scope, the use of network centrality algorithms computations could help to find the most influential players contributing to team performance and understand how the information is diffused [14, 15]. For example, teams with better performance outcome are associated with lower centralization, i.e., there is a lower dependency of the team from specific players [10]. Similarly, López Peña and Touchette [14] demonstrated that winning teams presented lower betweenness scores, i.e., higher fluidity of the ball exchanging dynamics between teammates. However, the abovementioned studies are exclusively focused on elite teams, therefore, there is a lack of studies describing how passing networks can provide information in youth developmental age groups. Such information may afford important insights to elaborate normative behaviour models of collaborative work according to the different development stages. Also, it allows to understand if the team network distribution is sustained by the offensive principles, which in turn help coaches to guide their intervention. Moreover, it may help to identify the key players involved during the offensive process and to describe their predominance of linkages within team positioning strategy at different levels of analysis [16, 17].

Although insightful information can be derived from the previous mentioned performance indicators and passing networks, the method only provides a discrete description of players’ behaviours. These studies’ outcomes are most likely based on consequences of performance, and therefore, the information about performances’ causes are very residual because it is given only a partial picture of the team’s behaviour. Thus, the notational analysis provide insufficient information to explain why and how players and teams regulate their performance in space and time to achieve a common goal [18]. In this sense, the players’ positioning in the pitch emerged recently as one of the key determinants of football performance [19, 20], once its linked to the players’ spatial-temporal relationships in respect of the collective principles of play. In fact, positioning-derived variables are now being widely used to measure the tactical performance while maintaining situational and sequential game characteristics [21, 22]. These studies use frequently non-linear parameters, such as Approximate Entropy (ApEn), to identify the players’ movement regularity behaviour [21, 23, 24]. For example, Gonçalves and colleagues [21] used the ApEn computations to identify that players’ positions are related to each position-specific centroid (defenders, midfielders or forwards) during a simulated 11-a-side association football game. Other positioning-derived variables such as teammates dyads positioning synchronization, absolute distance and distance variability [25, 26], Voronoi diagrams computations [27], major ranges positioning within distances of team centroids to goals and distances between the teams' opposing line-forces [28], team stretch index and speed of spread [29], have been used to help measuring football tactical behaviour.

Considering the couple of the strengths from both network approach and positioning-derived variables, it may be possible to create an association between the dynamical tactical relations stablished among teammates and the environment, as well as with the in-game decision’ consequences. Following this insight, the present study aims to explore how passing networks and positioning variables can be linked to the match outcome in youth elite association football. Understanding how youth football players base their game interactions may constitute a solid criterion for fine-tuning the training process and, ultimately, to achieve better individual and team performances during competition. Considering that tactical knowledge is learned and refined, it is essential to formulate comprehensive training processes for youth athletes [30]. This approach incorporates a wide range of complex individual behaviours and explores how these may affect and be affected by the group structures. Concomitantly, it may afford a holistic overview from the team performance as a systemic unit of analysis, while take into consideration the qualities of individuals in the team.

## Methods

### Participants

The participants were 44 male elite young Portuguese association football players from under-15 and under-17 age groups (U15: n = 22, age 13.9±0.3 years, height 1.69±0.07 m, weight 59.1±5.4 kg and playing experience 5.3±1.6 years; U17: n = 22, age 15.7±0.5 years, height 1.75±0.05 m, weight 66.4±3.5 kg and playing experience 7.4±1.3 years). The players were part of the same sports club and both age groups were competing in the national championship. The goalkeepers’ positioning it is very restricted to a specific area and their positioning dynamics are different from the outfield players. For this reason, the goalkeepers participated in the study but were excluded from the analysis. All participants, their parents, and the teams’ supervisors were informed about the research procedures, requirements, benefits and risks, and their written consent was obtained before the study began. The study protocol was approved and followed the guidelines stated by the Ethics Committee of the Research Centre for Sport Sciences, Health and Human Development, based at Vila Real (Portugal) and conformed to the recommendations of the Declaration of Helsinki.

### Procedures

Before the beginning of each session, players were outfitted with a 5Hz non-differential global positioning system (SPI-ProX, GPSports, Canberra, ACT, Australia) to record the dynamical positional data. The validity and reliability of these devices has already been inspected by independent verifications. The typical error of measurement is below 5% when accounting for measurement of the total distance covered, and between 5% to 10% during peak speed [31].

The testing session consisted of two friendly football games; one for the U15 and one for the U17, and both age groups followed the same protocol procedures. All players performed a 15-min standard warm-up consisting of a ball-possessing and dynamic stretching. Subsequently, two 11-a-side teams played a 50-min game (two periods of 25 min, interspersed by 10 min of passive recovery) on a 106×65 m artificial turf pitch, with official football rules. The total game duration was purposely manipulated to prevent the effects of fatigue in the studied variables [32]. It was indented to maintain the functional dependence between the player and performance context at the maximum potential. Both U15 and U17 head coaches distributed the players into two balanced teams according to the players’ pitch positions, physical performances and participation in competitive official matches [21]. Teams structure was a 1-4-3-3 formation, comprising 1 goalkeeper, 4 defenders, 3 central midfielders and 3 forwards, with these players assuming the standard behaviour of these positional roles [21, 33]. The session ended with a 10-min standardised cool-down, which consisted of low intensity running and static stretching exercises.

The matches were recorded with a standard digital camera (Sony CX625 Handycam®) located at ~15 m lateral/above of the pitch centre. The video files were downloaded to a computer and the number of successful passes performed between all possible outfield teammates dyads (45 teammates dyads with bidirectional possibility of pass = 90 dyads) and the number of shots were notated for each team. Also, the teams’ efficacy (efficacy = number of goals * 100 / number of shots) were reported to measure the team performance outcome. These performance indicators were gathered by two experience researchers in performance analysis and data reliability was inspected by retesting 15% of the sample one week later. The inter-class correlation coefficients were high (ICC > 0.94) [34].

### Passing network characterization

All networks representations and centrality based measures were calculated using Cytoscape® v3.1.1 [35, 36] with *CentiScaPe2*.*1* plugin [37, 38]. The software allowed to develop the teams’ passing networks, thus, the nodes represent players and connecting edges, were weighted exponentially according to the number of successfully passes performed between them. Afterwards, to identify the influence of a given player performance in a team-passing dynamic during game, the network centrality measures were computed for directed networks, which considers the direction of the pass, i.e., given two players A and B, it is possible that there are no passes (paths) between them during the game, or there is a pass (path) from node A and B but not from node B to node A. The closeness and betweenness centrality scores of the players within teams’ passing networks representation were computed and represented. These centrality measures were calculated based on the adjacency matrix where, say *v* and *w*, represents the number of passes from player *v* to player *w*. Also, the number of passes introduced in the matrix was used to measure the strength of a directed edge in the passing network and also to define a notion of geodesic distance (*dist(v*,*w)—*the length of the shortest path from player *v* to player *w*) [14, 37, 39]. Finally, the networks were shaped according to specific players positioning on the pitch illustration.

#### Closeness centrality

A closeness score indicates how easy it is for a player to be connected with teammates (by passing relation) and, therefore, that player is requested by the team as a target to pass the ball. Thus, it quantifies the proximity of how close is such player to his peers [40]. Closeness centrality is defined as the inverse of the farness, where higher values assume a positive meaning in the node proximity [8, 15, 39]. It is calculated by computing the shortest path between the node *v* and all other nodes, and then calculating the summa. Once this value is obtained, its reciprocal is calculated (see Scardoni, Petterlini and Laudanna [37] for complementary mathematical information):

$$Closeness\;(v)=\frac{1}{\sum_{w\in V}dist(v,w)}$$

#### Betweenness centrality

A player with higher betweenness scores is crucial to maintain team passing connections by acting as a connecting bridge. Also, low scores and spread across certain players may be related with well-balanced passing strategy and less specific players’ dependence [14, 41]. Betweenness centrality quantifies the occurrences that a node acts as a bridge along the geodesic path between other nodes [15, 39]. It is calculated based on couple of nodes (*v* and *w*, for instance) counting the total numbers of geodesic paths linking *v* and *w* and the number of those paths that intersect a node *n* (see Scardoni, Petterlini and Laudanna [37] for complementary mathematical information):

$$Betweenness(v)=\sum_{s\neq v\in V}\;\sum_{t\neq v\in V}\delta_{st}\;where\;\delta_{st}(v)=\frac{\sigma_{st}(v)}{\sigma_{st}}$$

$$\sigma_{st}\;is\;the\;number\;of\;shortest\;paths\;from\;node\;s\;to\;node\;t$$

$$\sigma_{st}(v)\;is\;the\;number\;of\;shortest\;paths\;from\;node\;s\;to\;node\;t\;passing\;for\;node\;v$$

#### Positioning relations

The positional coordinates from the players, i.e. latitude and longitude, were exported from the GPS units and computed using dedicated routines in Matlab (MathWorks, Inc., Massachusetts, USA) according to previous data filtering guidelines [26]. The obtained data was then used to calculate the distance between all possible dyads of outfield teammates (total of 45 dyads). Afterwards, the approximate entropy technique (ApEn) was performed to assess regularity in each one of the 45 dyads distances time series (regularity of the intra-team dyads positioning) [25]. The outcome range between 0 and 2 (arbitrary units) and lower values represented more repeatable, regular, predictable and less chaotic sequences of data points [42, 43]. From a processing approach, the ApEn expresses the probability that the configuration of one segment of data in a time series allows the prediction of the configuration of another segment of the time series from a certain distance apart [44]. In a practical perspective, this technique identifies if players’ dyads displacement trajectories express a regular and predictable pattern which may, in turn, provide information regarding their tactical behaviour [45, 46]. Afterwards, the players’ dominant region was obtained by the dynamic Voronoi cell computation, also using routines built in Matlab [27]. For each player, the mean area from entire game and corresponding coefficient of variation (CV) were computed and represented.

### Data analysis

A two-step cluster with log-likelihood as the distance measure and Schwartz’s Bayesian criterion was performed to classify the regularity in teammates dyads’ positioning (i.e., all ApEn dyads values) for each team. The analysis classified the dyads positioning into higher, medium and lower regularity. The number of shots, number of passes, closeness centrality and betweenness centrality values were compared between teams via standardized mean differences, computed with pooled variance and respective 90% confidence intervals [47]. Thresholds for effect size statistics were 0.2, trivial; 0.6, small; 1.2, moderate; 2.0, large; and >2.0, very large [47]. Smallest worthwhile differences were estimated from the standardized units multiplied by 0.2. Uncertainty in the true differences of the scenarios was assessed using non-clinical magnitude-based inferences with a specific statistical spreadsheet [48]. Magnitudes of clear effects were described according to the following scale: 25–75%, possible; 75–95%, likely; 95–99%, very likely; .99%, most likely [47]. The relationship between the number of passes performed and the positioning regularity values (across dyads) were assessed with Pearson’s product moment (*r*) and the following criteria used to interpret the magnitude of the correlation measures: ≤0.1, trivial; >0.1–0.3, small; >0.3–0.5, moderate; >0.5–0.7, large; >0.7–0.9, very large; and >0.9–1.0, almost perfect [47]. If the 90% CI overlapped positive and negative values, the magnitudes were considered unclear [49]. The two-step cluster analysis as well as Pearson’s product moment were carried in SPSS (IBM Corporation, USA).

## Results

**Fig 1:**
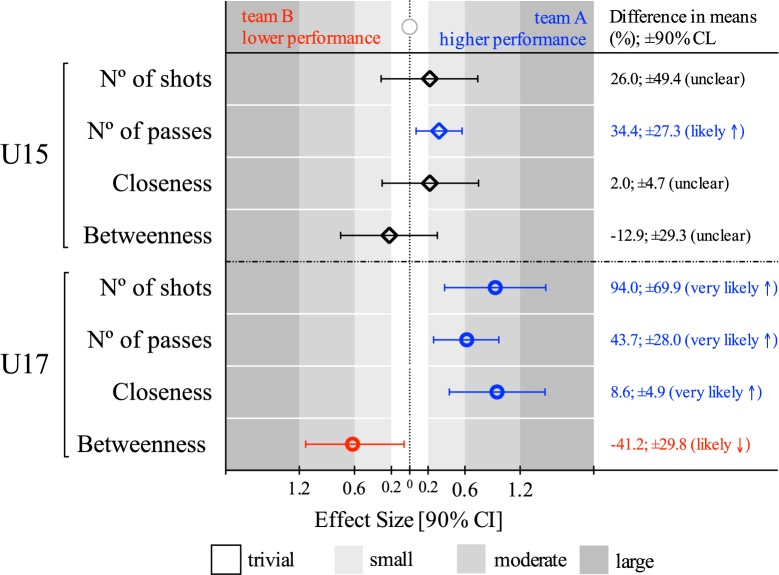
Between-teams’ differences for both age group analyses. Right side of the graph means higher values to the higher performance team in the match (U15 team A and U17 team A), left side more to lower performance team.

Teams were classified into lower and higher performance using the number of shots and teams’ efficacy. For the U15 game, the observed differences showed unclear tendencies in the number of shots, closeness centrality and betweenness centrality (team A, efficacy = 7.1%; Team B, efficacy = 10.1%). However, there was a small difference in the number of passes (34.4%; ±27.3%: mean difference %, ±90% confidence limits, likely), with higher values for the higher performance team. In the U17 game, the higher performance team (team A, efficacy = 33.3%) presented a moderate difference in the number of shots (94.0%; ±69.9%, very likely), number of passes (43.7%; ±28.0%, very likely), closeness (8.6%; ±4.9%, very likely) and betweenness (-41.2%; ±29.8%, likely) than the opponents (team B, efficacy = 14.3%) (see Fig 1).

**Fig 2:**
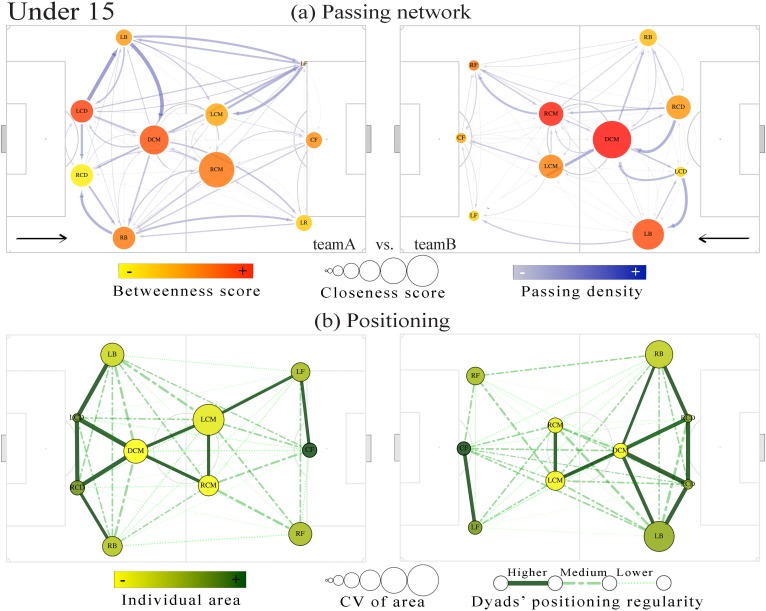
Visual representation from U15 match analysis. Upper panel, (a) passing network: nodes’ positions are represented by the player’s field position, sized according to the closeness centrality, and collared based on betweenness scores. The width of the edges grows exponentially with the number of passes successfully performed between two teammates and the colour density increase. Lower panel, (b) positioning: presents the dyads’ regularity classification; the regularity relations are represented based on cluster analysis. The nodes are collared according individual area and sized according to the corresponding CV.

**Fig 3:**
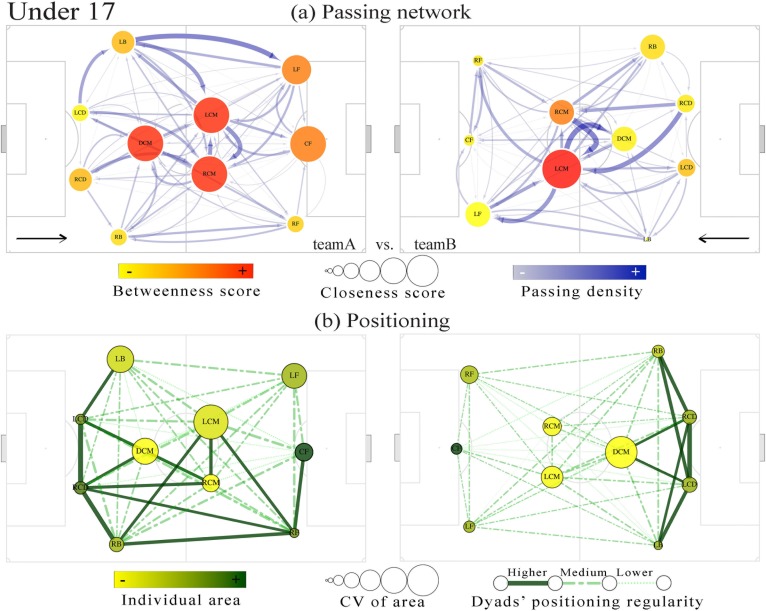
Visual representation from U17 match analysis. Upper panel, (a) passing network: nodes’ positions are represented by the player’s field position, sized according to the closeness centrality, and collared based on betweenness scores. The width of the edges grows exponentially with the number of passes successfully performed between two teammates and the colour density increase. Lower panel, (b) positioning: presents the dyads’ regularity classification; the regularity relations are represented based on cluster analysis. The nodes are collared according individual area and sized according to the corresponding CV.

Figs 2 and 3 present the overall representation from passing network and positioning relations established for each team. This visualization allows comparing the intra-team passing relation and the dyads’ level of regularity during the match. Concerning the U15 team with lower performance (team B), the defence central midfielder (DCM) presented higher importance in the network regarding both the closeness and the betweenness measures (Fig 2A, right networks), as well as the lateral central midfielder (LCM) of the lower performance team in U17 (Fig 3A, right network). However, in U17, the higher performance team presented higher centrality values distributed among the DCM, LCM and the right central midfielder (RCM) (Fig 3A, left networks). The cluster analysis showed that higher regularity in intra-team dyads positioning was mainly observed in defensive and midfield sectors and within the nearer teammates. Also, both central defenders and centre forwards presented higher individual area available to play and less variability, as expressed respectively by their Voronoi cells and their coefficient of variation (see Figs 2B and 3B).

**Fig 4:**
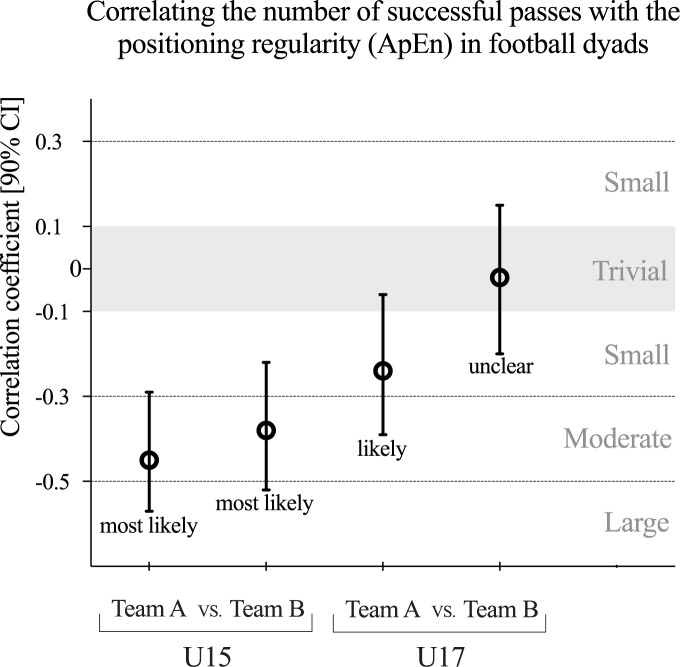
Teams’ correlation coefficient (with 90% confidence intervals) between the numbers of successfully passes performed and the positioning regularity values across dyads. Shaded area represents unclear correlation.

The relationship between the number of successful passes and the positioning regularity values is presented in Fig 4. There was a most likely negative moderate correlation between the number of passes and dyads’ positioning regularity for U15 game (team A: *r* = -0.44 [90% CI, -0.29; -0.58], team B: -0.38 [-0.22; -0.52]). For the U17 game, the team A presented a likely negative small correlation, but the team B correlation was unclear (-0.02 [-0.20; 0.15]). In both games, the correlation increased (in negative way) to the team who presented higher performance.

## Discussion

This study aimed to explore how passing networks and positioning variables can be linked to the match outcome in youth elite association football. The findings may provide insights to understand the reasons underpinning successful performances. It is suggested that lower passing dependency for a given player (lower betweenness scores) and higher intra-team well-connected passing relations (higher closeness scores) may optimize team performance.

The betweenness and closeness variables have been used previously to identify the importance and connectedness between players [10, 14]. In this study, an increase in the number of shots and passes as the closeness values increase and the betweenness values decrease has been found. Therefore, it is likely that a team lower betweenness may indicate a higher ability to maintain the ball flow, with less dependency on specific players [14]. Teams that are more dependent on specific players’ may be more easily blocked, since they show a lower balanced passing strategy distribution [50]. As so, teams with higher closeness and lower betweenness scores are more connected and seem to be less depending on the effort of a few players to pass the ball around [9, 14].

The findings of the current study also suggest that the team that presented higher passing density (i.e., number of passes successfully performed) achieved more successful shots. These results are in line with the available literature, showing that more goals are likely to be scored by teams with higher passing rate [50]. It seems relevant for coaches to be aware of how dependable they are from specific players and, also, the preferred passing teammates for each player and playing position. In this sense, by identifying players that are less required during the match, coaches could design training tasks to improve their technical-tactical skills and overcome the team’s dependency of specific players. In addition, this approach could also help to identify the level of experience and adherence to the game tactical principles from the studied teams, as the U17 teams presented a higher passing density and closeness scores than the U15 teams, showing a more balanced distribution of the network. Additionally, the interdependence of behaviours is commonly accepted as one of the most relevant characteristics of the complex adaptive systems [51]. Since these systems are considered as a group of independent individuals that act through synergies, the interdependence in team sports can be understood as a group of players (team) that need to cooperate to achieve shared goals [52]. Thus, the observable behaviours are extremely contextual-dependent [53] and consequently, the players’ decisions are dynamically constrained by the teammates and opponents [54]. Adding together, the team’s strong dependence on specific players will influence negatively the overall performance. In fact, it was already shown that the difference between teams’ performance it is related in the way of how their members interact between them [10]. Thus, if a team has a key player, it is likely that the game dynamics it is supported on the actions of that player, letting the team more vulnerable and dependent.

There is a lack of studies supporting coaches’ decisions when related to youth tactical principles. The available research is focused on identifying which learning environment may better help to develop the players' specific characteristics [55, 56]. In the current study, we intended to understand how the regularity positioning between teammates may be correlated with the number of passes performed. The positioning data can be used to measure tactical behaviour in football [46], since the players’ displacement behaviours may explain the level of intra-teams’ synchronization and coupling relations [21, 26]. Current findings identify higher regularity in positioning with the nearest teammates, mainly in defence/midfield pitch sectors. Apparently, the level of interpersonal coordination is influenced by the distance between players [26], therefore, it is likely that the players are more coupled with the players with similar tactical roles [21]. Gama and colleagues [57] showed that teammates’ behaviour interactions tended to appear during the offensive phase and mainly organised in the central and lateral areas of the pitch. Also, there seems to be a clear influence of opponents’ behaviour strategy to remove the most influence players from the central positions of the passing network [58] and also to maintain the balance from attacker-defender dyads positioning in all in-game situations [59]. Thus, the players’ behaviour is constrained by several variables, such the environmental information, the position of the teammates, the opponents, the goals, and the ball [21, 26, 60]. The results from this study identified a correlation between the dyads’ positioning regularity and the passing density. This evidence was stronger in the U15 game, and highlights that players’ positioning are more dependent on the ball position than the U17 teams. It is possible that due to the higher game knowledge, the U17 age group attuned more their position with teammates and opponents and less by the ball location. Interestingly, the correlation differences in both U17 teams could be related with the more robust values of closeness and lower of betweenness, comparatively to the U15 teams. This evidence might suggest that when the level of team dependency of specific players is lower, it is more likely that players positioning become coupled and less random, as it could happen in teams that adapt their position according to the best players’ actions. Also, it seems that the increase in the number of passes among both higher U15 and U17 teams strengthens the previous correlation since it is easier to maintain the positional regularity [41].

Nevertheless, further studies on this topic can extend the research by addressing several relevant questions. For example, there is a lack of information regarding to official youth matches since the positioning tracking systems have been used only in senior professional teams. This study intended to overcome this issue, however, formal competitive environments should be used in future studies to provide a step forward insight. The present exploratory data-approach should also be applied to a wide range of contextual variables, such as game status, different teams’ tactical formations, playing home vs. playing away, etc. These different contexts may afford different collective behaviours understanding which, in turn, will enrich the performance programs development.

The modelling network procedures allowed identifying different team’s passing dynamics structures and presented a visualisation method to represent the obtained structures. Consequently, it seems easy to identify the players with most important role in the passing networks and coaches can understand: (i) the demanded informational constraints that shape the players’ passing decisions; (ii) which players are being over-solicited during competition; (iii) weaknesses in the team specific positions, based on lower teammates passing relations. Coaches of youth age groups may use the information provided to improve their training tasks sessions and optimize the design of representative situations, where the passing lines are more diffused and unpredictable.

## Conclusions

In summary, this study provided evidence that a lower passing dependency for a given player and higher intra-team well-connected passing relations may optimize team performance. Regarding the correlation between the dyads’ positioning regularity and the passing density, it was found a higher correlation in U15 indicating a possible more dependence of the ball position rather than in the U17 teams. This study emphasized the potential of coupling notational analyses with spatial-temporal relations to produce a more functional and holistic understanding of teams’ sports performance. The main findings have several implications for the training process design, particularly by helping to identify the major features of youth football teams’ behavioural dynamics. Also, this study extends the base of scientific knowledge related to the power of social network analysis approach, by providing novel insight into group structures performance. Within, the overall groups’ outcome is strongly related with the interdependence from teammates and the way they dynamically co-adapt their task-roles.
